# Molecular and Phenotypic Characteristics of Healthcare- and Community-Associated Methicillin-Resistant *Staphylococcus aureus* at a Rural Hospital

**DOI:** 10.1371/journal.pone.0038354

**Published:** 2012-06-15

**Authors:** Amy E. Peterson, Meghan F. Davis, Kathleen G. Julian, Grace Awantang, Wallace H. Greene, Lance B. Price, Andrew Waters, Avanthi Doppalapudi, Lisa J. Krain, Kenrad Nelson, Ellen K. Silbergeld, Cynthia J. Whitener

**Affiliations:** 1 Johns Hopkins Bloomberg School of Public Health, Baltimore, Maryland, United States of America; 2 Penn State Hershey Medical Center, Hershey, Pennsylvania, United States of America; 3 The Translational Genomics Research Institute, Flagstaff, Arizona, United States of America; Rockefeller University, United States of America

## Abstract

**Background:**

While methicillin-resistant *Staphylococcus aureus* (MRSA) originally was associated with healthcare, distinct strains later emerged in patients with no prior hospital contact. The epidemiology of MRSA continues to evolve.

**Methods:**

To characterize the current epidemiology of MRSA-colonized patients entering a hospital serving both rural and urban communities, we interviewed patients with MRSA-positive admission nasal swabs between August 2009 and March 2010. We applied hospitalization risk factor, antimicrobial resistance phenotype, and multi-locus sequence genotype (MLST) classification schemes to 94 case-patients.

**Results:**

By MLST analysis, we identified 15 strains with two dominant clonal complexes (CCs)–CC5 (51 isolates), historically associated with hospitals, and CC8 (27 isolates), historically of community origin. Among patients with CC5 isolates, 43% reported no history of hospitalization within the past six months; for CC8, 67% reported the same. Classification by hospitalization risk factor did not correlate strongly with genotypic classification. Sensitivity of isolates to ciprofloxacin, clindamycin, or amikacin was associated with the CC8 genotype; however, among CC8 strains, 59% were resistant to ciprofloxacin, 15% to clindamycin, and 15% to amikacin.

**Conclusions:**

Hospitalization history was not a strong surrogate for the CC5 genotype. Conversely, patients with a history of hospitalization were identified with the CC8 genotype. Although ciprofloxacin, clindamycin, and amikacin susceptibility distinguished CC8 strains, the high prevalence of ciprofloxacin resistance limited its predictive value. As CC8 strains become established in healthcare settings and CC5 strains disseminate into the community, community-associated MRSA definitions based on case-patient hospitalization history may prove less valuable in tracking community MRSA strains.

## Introduction

Methicillin-resistant *Staphylococcus aureus* (MRSA) is one of the most commonly detected antimicrobial-resistant pathogens globally and is a major public health concern in the United States [1], [2]. Among *S. aureus* isolates from a U.S. network of over 300 microbiology laboratories, the number resistant to methicillin nearly doubled between 1999 and 2006, with over 50% of *S. aureus* strains from both inpatients and outpatients identified as methicillin-resistant in 2006 [1].

The epidemiology of this pathogen, originally associated with hospitals, is changing rapidly [3]. In the 1990s, distinct strains emerged in patients with no prior hospital contact [4], [5]. The prevalence of such community-associated (CA-)MRSA strains has been increasing over time [1], [3], [6]. In 2004, MRSA nasal colonization was estimated at 1.5% of the U.S. population, or approximately 4 million people [7]. Nasal colonization increases risk for later development of MRSA infection [8].

Isolates from patients with community-associated MRSA historically demonstrate several important differences compared to healthcare-associated strains, including molecular differences identified by typing methods, *e.g.* pulsed-field gel electrophoresis (PFGE) [9] or multi-locus sequence typing (MLST) [10]. For example, the USA300/CC8 strains that are most commonly associated with community transmission cycles often contain the virulence factor Panton Valentine leukocidin (PVL) cytotoxin and are more likely to be susceptible to certain antimicrobial agents commonly used to treat MRSA, particularly clindamycin [10]–[12] and aminoglycosides [11], [13]. Recently, these distinctions have begun to blur as community strains become “domesticated” to the hospital and healthcare settings [11], [14]–[18] and hospital strains “go feral” and establish in the community [19]. Despite changes in epidemiologic patterns by molecular type, some authors have suggested that antimicrobial susceptibility may continue to be a distinguishing characteristic of community-acquired MRSA [10]–[12].

To better understand the current epidemiology of CA-MRSA in a population that includes rural communities, we conducted a study of MRSA nasal colonization of patients at the time of admission to Penn State Hershey Medical Center (PSHMC), a 500-bed tertiary care center in south-central Pennsylvania.

## Methods

### Ethics Statement

The Johns Hopkins Bloomberg School of Public Health and Penn State Hershey Medical Center Institutional Review Boards reviewed and approved this study. Patients gave written informed consent for participation in the study.

### Hospital Surveillance

As part of an ongoing hospital surveillance program, nasal swabs were performed on all patients admitted to PSHMC, except those in the obstetrical wing, who did not have a known prior diagnosis of MRSA.

### Research Design

As part of a study designed to evaluate risk factors for MRSA nasal carriage related to household and rural exposures, we approached patients admitted between August 2009 and March 2010. Patients who were over 18 years of age and were screened for MRSA on admission were eligible to participate in our study. After obtaining informed consent, we interviewed both positive and negative MRSA case-patients. This manuscript is limited to the analysis of the risk factors reported by patients identified with a positive nasal swab and isolates from those case-patients that were subsequently confirmed positive by culture and molecular methods, strain-typed by MLST, and tested for antimicrobial susceptibility.

### Survey

Patients were interviewed for self-reported risk factors that included location of residence, demographic information, and hospitalization within the past month, six months, or year prior to admission. Epidemiologic categorization of MRSA isolates from case-patients as “healthcare-associated” was based on answers of “yes” to “Have you been hospitalized at any point in the past a) year, b) six months, or c) one month?” and we defined patient hospitalization exposure as an admission to a healthcare setting lasting eight hours or longer. Patient records were reviewed for long-term healthcare residence immediately prior to hospital admission, and patients with such residence also were classified as HA-MRSA case-patients. History of antimicrobial use, which has been included in some alternative definitions of healthcare-associated MRSA, was left out of our definition to allow it to be examined separately.

### Sample Collection

Swabs of the anterior nares of patients were collected within 48 hours of admission. These swabs were processed using the BD GeneOhm™ MRSA Assay (Becton Dickinson Diagnostics, San Diego, CA). This method allows for direct detection of MRSA from the nasal specimen and has a manufacturer-reported sensitivity of 93% and a specificity of 96%. This PCR-based assay detects unique gene sequences for identification of both *S. aureus* (*orfX*) and of methicillin resistance (SCCmec) [20], [21]. Positive nasal swabs were inoculated into trypticase soy broth (TSB) +20% glycerol and archived at −80°C.

All available PCR-positive nasal swabs from patients enrolled in the study were cultured for MRSA. A 10 µl loop of broth from each archived nasal swab was plated onto commercial MRSA Select™ agar plates (Bio-Rad Laboratories, Hercules, CA) and incubated for up to 48 hours at 35°C [22]. Archived swabs that did not demonstrate positive growth on a first culture attempt were inoculated into enrichment broth (Mueller-Hinton broth +6.5% NaCl) and incubated for 24 hours at 35°C. From the overnight enrichment, a second attempt was made to isolate positive cultures on MRSA Select™ agar.

### Molecular Methods

DNA was extracted from isolates using ZR-96 DNA extraction kits (Zymo Research, Orange, CA). Isolates were tested using a real-time PCR assay to confirm MRSA genotype by detection of *mecA* and *femA* genes (Pathogene, LLC). Isolates positive for *femA* but negative for *mecA* (MSSA) on real-time PCR were re-tested using a new universal *mecA* primer as previously described [23]. Additionally, isolates were tested for the Panton Valentine leukocidin toxin using a real-time PCR assay for the *luk_PV_SF* region as described previously [24].

Confirmed MRSA isolates were typed by multi-locus sequence typing (MLST) as described previously [25]. Sequences from seven housekeeping genes were compared to an online database (www.mlst.net) to identify a match for each allele at that locus, allowing strains to be assigned a numerical strain type (ST) based on the allelic profile. Using eBURST software (eburst.mlst.net), related clusters of MRSA sequence types were grouped into Clonal Complexes (CC), which consist of a main or progenitor ST and other STs that differ by one or two alleles [25], [26].

### Antimicrobial Susceptibility Testing

Confirmed MRSA isolates were tested subsequently for antimicrobial susceptibility using disc diffusion methods [27], [28], including erythromycin-induced resistance to clindamycin (D-test), following CLSI guidelines [29]. Antibiogram classifications were made on the basis of susceptibility to seven antimicrobials: tetracycline (Te), gentamicin (GM), amikacin (AN), trimethoprim/sulfamethoxazole (SXT), clindamycin (CC), ciprofloxacin (CIP), and erythromycin (E). Multi-drug resistance (MDR) was defined as beta-lactam resistance by *mecA* gene presence plus nonsusceptibility (inducible, intermediate or high-level resistance) to three additional classes of antimicrobials by disc diffusion methods, based on a definition reported by SENTRY [30].

### Statistical Analysis

We estimated associations between hospital risk factor, phenotypic, and genotypic classification methods using prevalence ratios. We calculated estimates of association (prevalence ratios) using Poisson models with robust estimation of standard errors as described previously [31], [32] using Stata 11 (College Station, TX). We evaluated the effect of changing the temporal cutoff for our criteria of self-reported hospitalization on estimates of association for our results based on hospital risk factor-based classification. Reference groups were assigned to the largest strata.

## Results

### Case-patient Selection

We approached 657 patients at Penn State Hershey Medical Center (PSHMC) between August 2009 and March 2010. Of these, 408 (62%) consented, including 151 MRSA screen- positive case-patients and 257 screen-negative case-patients. Of the 151 case-patients, 94 had isolates confirmed as MRSA available for MLST analysis and antimicrobial susceptibility testing, and therefore were included in this analysis.

Epidemiologic comparison of the 57 case-patients for whom MRSA isolates were not available demonstrated that these patients did not differ significantly in demographic characteristics, rates of prior hospitalization, or self-reported antimicrobial use as compared to the 94 patients for whom isolates were available for further genotypic and phenotypic analysis.

### MLST Genotypes

The most common sequence type was ST5 (34 isolates). Fifty-one isolates (55%) were associated with clonal complex 5 (CC5), which includes ST5, ST225, and the third most common type, ST105 (11 isolates). Twenty-seven isolates (29%) were associated with clonal complex 8 (CC8), which includes ST72 (1 isolate) and the second most common type, ST8 (26 isolates). Additional sequence types included: ST1 (*n* = 5), ST15 (*n* = 2), ST30 (*n* = 1), ST59 (*n* = 1), ST81 (*n* = 1), ST221 (*n* = 1), ST474 (*n* = 1) & ST496 (*n* = 1). A ST could not be assigned to three isolates; one of these isolates was determined to be a novel sequence, one was non-typable, and one, originally negative for *mec*A on qPCR, never was tested by MLST but later was found to be positive for *mec*A using a universal *mec*A primer according to Holden *et al.* [23]. CC8 and CC5 strains were isolated from case-patients with similar age and gender characteristics. Case patients with CC8 isolates were less likely than case patients with CC5 isolates to report recent antimicrobial use (PR 0.97 for one to six months prior use, and PR 0.53 for use within the past month), but these estimates of association were not statistically significant (*p* = 0.93 and *p* = 0.10, respectively).

### Antimicrobial Susceptibility Phenotypes & Pvl Gene Presence

**Figure 1:**
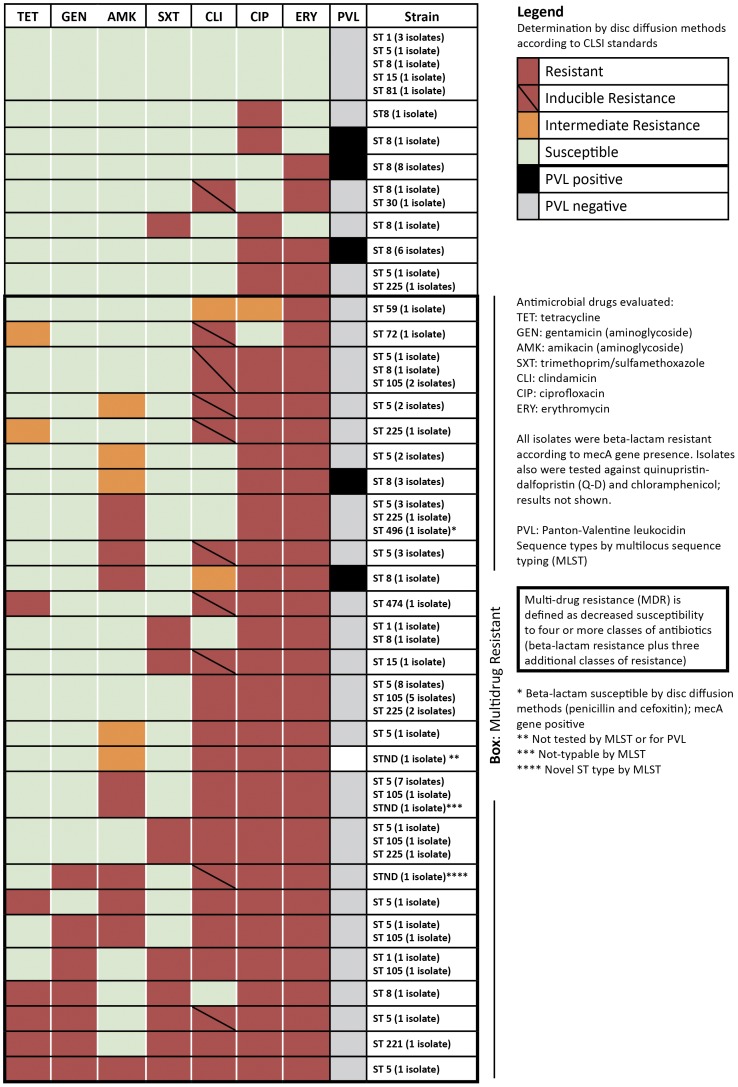
Heat map of antimicrobial susceptibility patterns for 94 methicillin-resistant *Staphylococcus aureus* (MRSA) strains from Penn State Hershey Medical Center, August 2009 to March 2010.

We identified 30 antimicrobial susceptibility profiles among the 94 isolates representing 15 different ST types (**Figure 1**). Only seven isolates were pan-susceptible to the tested antimicrobials, and one was resistant to all antimicrobials tested. Among case-patients without any history of hospitalization, three (9%) were nonsusceptible to tetracycline, two (6%) to trimethoprim-sulfamethoxazole and 18 (55%) to clindamycin. Of 94 MRSA isolates tested, 66 (70%) overall were nonsusceptible to ≥4 classes of antimicrobials (MDR). All *pvl*-positive isolates were ST8 (CC8) strains. Of the 19 *pvl*-positive isolates, 4 (21%) were MDR.

### Classification as HA- or CA-MRSA by Hospitalization Risk Factor

Among patients with CC5 isolates, 25% reported no history of hospitalization in the past year and 43% reported no history of hospitalization within six months of admission (**Table 1**). Among patients with CC8 isolates, 59% reported being hospitalized in the past year, and 33% reported being hospitalized within six months.

**Table 1:** Impact of changing the cutoff point of history of hospitalization from within prior year to within prior six months or one month on classification as HA-MRSA vs. CA-MRSA according to strain (ST) and clonal complex (CC) assignment from multi-locus sequence typing (MLST) and according to multi-drug resistance phenotype.

Hospitalization	In the past year (HA)	Not in the past year (CA)	In the pastsix months (HA)	Not in the pastsix months (CA)	In the pastmonth (HA)	Not in the past month (CA)
**Genotype (** ***n*** ** = 78)**						
**All CC8 (** ***n*** ** = 27)**	**16 (59%)**	**11 (41%)**	**9 (33%)**	**18 (67%)**	**5 (19%)**	**22 (81%)**
ST8 (*n* = 26)	16 (62%)	10 (38%)	9 (35%)	17 (65%)	5 (19%)	21 (81%)
**All CC5 (** ***n*** ** = 51)** *(reference)*	**38 (75%)**	**13 (25%)**	**29 (57%)**	**22 (43%)**	**19 (37%)**	**32 (63%)**
ST5 (*n* = 34)	25 (74%)	9 (26%)	18 (53%)	16 (47%)	12 (35%)	22 (65%)
ST105 (*n* = 11)	9 (82%)	2 (18%)	8 (73%)	3 (27%)	5 (45%)	6 (55%)
**PR** b **for CC8 (CA vs HA)**	**1.59**	*ref*	**1.55**	*ref*	**1.30**	*ref*
**[95% CI]**	[0.83–3.09]	*ref*	[1.02–2.34]	*ref*	[0.98–1.72]	*ref*
***p-value***	*p = 0.16*	*ref*	*p = 0.04*	*ref*	*p = 0.07*	*ref*
**Phenotype (** ***n*** ** = 94)**						
**Not MDR (** ***n*** ** = 28)**	15 (54%)	13 (46%)	7 (25%)	21 (75%)	5 (18%)	23 (82%)
**MDR (** ***n*** ** = 66)** *(reference)*	46 (70%)	20 (30%)	38 (58%)	28 (42%)	22 (34%)	43 (66%)
**PR for susceptibility (CA vs HA)**	**1.53**	*ref*	**1.76**	*ref*	**1.24**	*ref*
**[95% CI]**	[0.89–2.64]	*ref*	[1.24–2.52]	*ref*	[0.97–1.59]	*ref*
***p-value***	*p = 0.12*	*ref*	*p = 0.002*	*ref*	*p = 0.09*	*ref*

Values are *n* (%). Multi-drug resistance is defined as non-susceptibility beta-lactam drugs plus three or more classes of antimicrobials. Beta-lactam resistance is determined by the presence of the *mecA* gene.

a Missing information for hospitalization within one month for 1 individual. This individual had a non-CC8, non-CC5 isolate that was multi-drug resistant and is excluded from analysis by isolate phenotype.

b PR: Prevalence Ratio.

We examined the effects of varying cutoff criteria for hospitalization risk factor-based classification by assigning cases as epidemiologically HA-MRSA versus CA-MRSA based on self-reported patient histories of hospitalization within one year, six months, or one month of admission (**Table 1**). If cases were classified as epidemiologically HA-MRSA using the cutoff of hospitalization within the past six months, then there was an association with both CC5 genotype (PR 1.71, *p* = 0.08) and MDR (nonsusceptibility to ≥4 antimicrobials) profile (PR 2.34, *p* = 0.01). (Table 1 provides this comparison using the larger CC5 group as the reference instead.) Estimates of association were similar but non-significant when cutoffs were used of hospitalization within the past month or within the past year. However, using genotype as a gold standard, a negative history of hospitalization within six months provided a predictive value of only 45% for CA-MRSA and a positive history of hospitalization provided a predictive value of 76% for HA-MRSA in our study population. Changing the history of hospitalization cutoff provided similar predictive values for one-month and one-year time frames.

We examined whether presence of the Panton-Valentine leukocidin gene (*pvl*) was associated with epidemiologic classification. MRSA case-patients without a history of hospitalization within one year, six months, or one month were 1.67, 1.56, or 1.24 times more likely than HA-MRSA case patients to be colonized with *pvl*-positive isolates, respectively; however, only the estimate of association for the six-month cutoff was statistically significant (*p* = 0.07, *p* = 0.02, or *p* = 0.09, respectively).

### Sensitivity Analysis with Age Restriction

Because of our interest in community-associated MRSA, we performed a sensitivity analysis to examine estimates of association for a subset of case-patients by restricting models to participants who were under the age of 65. This removed the older population more likely to have recent indirect healthcare contact, *e.g.* home care or spousal residence in a nursing home. MRSA case-patients under the age of 65 without long-term care home residence or history of hospitalization within one year, six months, or one month were 2.43, 1.53, or 1.18 times more likely to be CC8 compared to CC5 genotypes, respectively (*n* = 45); however, only the estimate of association for the one-year cutoff was statistically significant (*p* = 0.05, *p* = 0.11, or *p* = 0.35, respectively). These CA-MRSA cases also were 2.24, 1.62 or 1.21 times more likely to be non-MDR, respectively (*n* = 53), but only the estimates of association for the one-year and six-month cutoffs were statistically significant (*p* = 0.02, *p* = 0.03, or *p* = 0.23, respectively).

### Antimicrobial Susceptibility Profiles by MLST Type

**Table 2** presents prevalence of susceptibility by antimicrobial according to ST type and clonal complex. Compared to CC5 isolates, CC8 isolates had a 3.78 [95% CI: 2.38–6.00] fold higher prevalence of ciprofloxacin susceptibility (*p*<0.001); a 9.72 [95% CI: 3.71–25.46] fold higher prevalence of clindamycin susceptibility (*p*<0.001); and a 3.22 [95% CI: 1.23–8.43] fold higher prevalence of amikacin susceptibility (*p* = 0.02). CC8 isolates had a 6.05 [95% CI: 3.10–11.78] fold higher prevalence of susceptibility to four or more classes of antimicrobials, *i.e.,* non-MDR (*p*<0.001).

**Table 2:** Prevalence of susceptibility to seven antimicrobials, by strain (ST) and clonal complex (CC), among 94 isolates.

	ST 5 (CC5) (*n* = 34)	ST 105 (CC5) (*n* = 11)	All CC5 (*n* = 51)	ST 8 (CC8) (*n* = 26)	All CC8 (*n* = 27)	*p-value*	All isolates (*n* = 94)
Erythromycin (ERY)	1 (3%)	0 (0%)	1 (2%)	5 (19%)	5 (19%)	*0.001*	10 (11%)
Ciprofloxacin (CIP)	1 (3%)	0 (0%)	1 (2%)	10 (38%)	11 (41%)	*<0.001*	18 (19%)
Clindamycin (CLI)	5 (15%)	0 (0%)	6 (12%)	23 (88%)	23 (85%)	*<0.001*	36 (38%)
Amikacin (AMK)	13 (38%)	9 (82%)	27 (53%)	22 (85%)	23 (85%)	*0.02*	62 (66%)
Trimethoprim/sulfamethoxazole (SXT)	31 (91%)	9 (82%)	45 (88%)	23 (88%)	24 (89%)	*0.93*	81 (86%)
Gentamicin (GEN)	31 (91%)	9 (82%)	46 (90%)	25 (96%)	26 (96%)	*0.41*	85 (90%)
Tetracycline (TET)	31 (91%)	11 (100%)	48 (94%)	25 (96%)	25 (93%)	*0.95*	86 (91%)

Values are *n* (%) for susceptibility (excluding isolates with inducible, intermediate, or high-level resistance).

P-values are reported for comparisons between CC8 and CC5 (*reference*).

CC5 includes ST5 (*n* = 34), ST105 (*n* = 11) and ST225 (*n* = 6). CC8 includes ST8 (*n* = 26) and ST72 (*n* = 1).

### Geographic Classification

Eight of 94 case-patients (9%) reported residence in the city of Harrisburg, one reported out-of-state residence, and two did not report location of residence. The remaining 83 case-patients (88%) reported non-urban residence in Pennsylvania, with 25 (27%) reporting residence in rural counties (*i.e.,* population density less than 284 persons per square mile, county data from www.rural.palegislature.us/rural_urban.html). We did not find significant associations between 2010 Census county population density or rural county residence and prevalence of strains by genotype, multidrug resistance, or hospital risk factor classification (results not shown).

## Discussion

In this study, we found that classification by history of hospitalization did not correlate well with the genotypes of MRSA that historically had emerged in association with hospitals (*e.g.,* CC5 complex, associated with PFGE type USA100), and that absence of hospitalization history also did not correlate with those genotypes that historically had emerged in patients without the association with healthcare (*e.g.,* CC8 complex, associated with PFGE type USA300). We did not test for SCC*mec* types, which may vary within MLST strain type; this is a limitation of our study. Our data indicate a shift in the epidemiology of CC8 strains such that a majority of these strains were isolated from patients who report a history of hospitalization, although a statistical trend remained for community association with CC8 strains and strains carrying the *pvl* gene. Conversely, a quarter of CC5 strains in our cohort came from patients without recent hospitalization. This possible shift could be due to circulation of strains of community origin in the hospital, and, to a lesser extent, dissemination of strains of hospital origin into the community.

These findings are consistent with recent reports of MRSA epidemiology in the United States [6], [15], [18], [33], [34] and internationally [14], [16], [17], [19]. Among children admitted in 2007 to the PICU at Johns Hopkins Hospital (which, like our study site, is a mid-Atlantic tertiary care center), CA-MRSA (USA300) isolates were found on admission (nasal colonization) and also isolated from hospital-associated MRSA infections [15]. Similar trends for USA300 strains have been seen among healthcare-associated bloodstream infections in the U.S. [11], [34], healthcare-associated infections internationally [35], and in a national Active Bacterial Core surveillance system [36].

The definition we used for HA- versus CA-MRSA is similar to common definitions used in the literature. Naimi *et al.* have defined HA-MRSA based on criteria of prior MRSA diagnosis, MRSA cultured on a test performed more than 48 hours after hospital admission (both not applicable in our study by design), history of hospitalization, surgery, dialysis or residence in a long-term care facility within a year before the culture date, or a permanent device such as an indwelling catheter present at the time of culture [37]. Analysis of our epidemiologically-based classification of HA-MRSA versus CA-MRSA cases suggests that a cutoff of hospitalization within six months rather than one year may be more consistent with isolate genotypic and antimicrobial phenotypic characteristics; the association, however, was not strong. This finding may reflect nasal persistence of strains or host characteristics within our cohort. For example, a European study found that, among patients colonized with MRSA, the median duration of colonization was seven months [38].

Multidrug resistance and resistance to ciprofloxacin, clindamycin, and amikacin significantly differentiated strains by genotype, with CC5 isolates showing generally more frequent resistance and CC8 isolates showing more frequent susceptibility to these three antimicrobials. High rates of ciprofloxacin resistance, even among isolates from CA-MRSA case-patients, may limit future discriminatory performance for this antimicrobial in this and similar patient populations. Results are consistent with clindamycin susceptibility reported in U.S. MRSA isolates with community genotypes (USA 300/CC8) [39] or community SCC*mec* types (SCC*mec* IV) [40].

Multidrug resistance (nonsusceptibility to ≥4 classes of antimicrobials) was highly prevalent in MRSA isolates from this PSHMC cohort. The overall rate of MDR was 70%, and even among historically-susceptible CC8 isolates, the rate of MDR was 30%. This generally is higher than earlier reports in the United States [10], [41], [42]. Within MLST type, strains were not uniform by antimicrobial susceptibility phenotype (antibiogram). While MLST designation relates to evolutionary MRSA lineage [43], antibiogram differences within strain likely reflect more recent genetic divergence characterized by acquisition of resistance.

This descriptive, observational study has several limitations. First, our results are restricted to comparisons within culturable isolates, and by necessity excludes 57 (38% of 151) MRSA case-patients whose nasal swabs were not available for culture as well as those whose presumptive MRSA-positive swabs did not grow on selective media. The latter may represent a source of selection bias for hardier strains or represent a bias favoring case patients with higher concentrations of nasal bacterial colonization more amenable to culture. Second, because we studied a single hospital inpatient cohort, results may not be generalizable to other adult populations. Third, we did not collect information by interview on all sources of potential exposure to healthcare settings, including dialysis, which could result in misclassification of cases as community- rather than healthcare-associated. However, an analysis restricted to participants aged 18–65, excluding the elderly case-patients who may have distinct risk factors such as unreported non-hospital healthcare facility or home care nursing contact, did not strongly influence the estimates of association. Finally, because risk factors were based on self-report, recall bias may result in misclassification, *e.g.,* by the hospitalization history classification method.

This study is strengthened by use of a hospital-wide surveillance program rather than selective risk-based testing for case-patient ascertainment. Because of the nature of the health care facility, it contributes to the literature on MRSA epidemiology by including participants from rural communities.

These results indicate discordance between patient risk factor epidemiology in relation to the MRSA isolates’ genotypic and antimicrobial resistance characteristics. The ever-increasing shift of healthcare from acute hospitals to non-acute and home settings may in part explain the findings. As CA-MRSA strains enter healthcare settings, and HA-MRSA strains disseminate to the community, isolate genotype and antimicrobial susceptibility phenotype provide different pieces of information to track movement of strains and inform community clinical practice and hospital infection control efforts.
